# Editorial Amenities

**Published:** 1883-01

**Authors:** 


EDITORIAL AMENITIES.—Without the knowledge of the Dental Editor of this journal, the *Cosmos* was given an opportunity to perform a usual act of courtesy, which it accomplishes in its own peculiar manner. We acknowledge the hit, and might retort with a *tu quoque;* but this would be somewhat descending from the higher level which we assume and are resolved to maintain. We can therefore smile benignantly upon our ironical *confrere.*

MEDICAL EXCHANGES, articles for publication and books for notice in the Medical Department must be addressed to the "Medical Editor, 411 Lexington Avenue, New York City."

DENTAL EXCHANGES, articles for publication and books for notice in the Dental Department must be addressed to the "Dental Editor, 11 West Chippewa street, Buffalo, N. Y."

